# Infrared Illumination-Induced Responsivity Enhancement in Photoelectric Tunable-Step Terahertz Detectors

**DOI:** 10.1021/acsphotonics.6c01501

**Published:** 2026-09-04

**Authors:** Ran Chen, Matthew Tan, Harvey E. Beere, Jonathan P. Griffiths, David A. Ritchie, Wladislaw Michailow

**Affiliations:** † Cavendish Laboratory, 2152 University of Cambridge, CB3 0HE Cambridge, U.K.; ‡ Center of Ionic Liquids and Green Energy, Beijing Key Laboratory of Solid State Battery and Energy Storage Process, Institute of Process Engineering, Chinese Academy of Sciences, Beijing 100190, China; § Centre for Integrative Semiconductor Materials, Swansea University, SA1 8EN Swansea, U.K.; ∥ Department of Physics, Swansea University, SA1 8EN Swansea, U.K.

**Keywords:** infrared illumination, persistent photoconductivity effect, terahertz detection, photoelectric tunable-step detectors, AlGaAs/GaAs heterostructures

## Abstract

Quantum phenomena in two-dimensional electron gases (2DEGs) provide new opportunities for high-responsivity far-infrared detection. A prime example is photoelectric tunable-step (PETS) terahertz (THz) detectors, which use the in-plane photoelectric (IPPE) effect as a sensitive photonic detection mechanism. Here, we show that the terahertz sensitivity of this quantum photodetection mechanism can be dynamically tuned by using infrared illumination. We leverage the persistent photoconductivity (PPC) effect in AlGaAs/GaAs-based heterostructures to tune the device performance by adjusting the electron density and mobility of the channel. Our approach enables optimization of the responsivity for different operating modes and dynamic control over the output impedance of PETS terahertz detectors of approximately one order of magnitude. We provide an explanation for the underlying physics that advances the understanding of quantum photodetection phenomena in 2DEGs. Our findings pave the way to in situ tunable optoelectronics combining concepts from the infrared and terahertz spectral regions.

## Graphical abstract

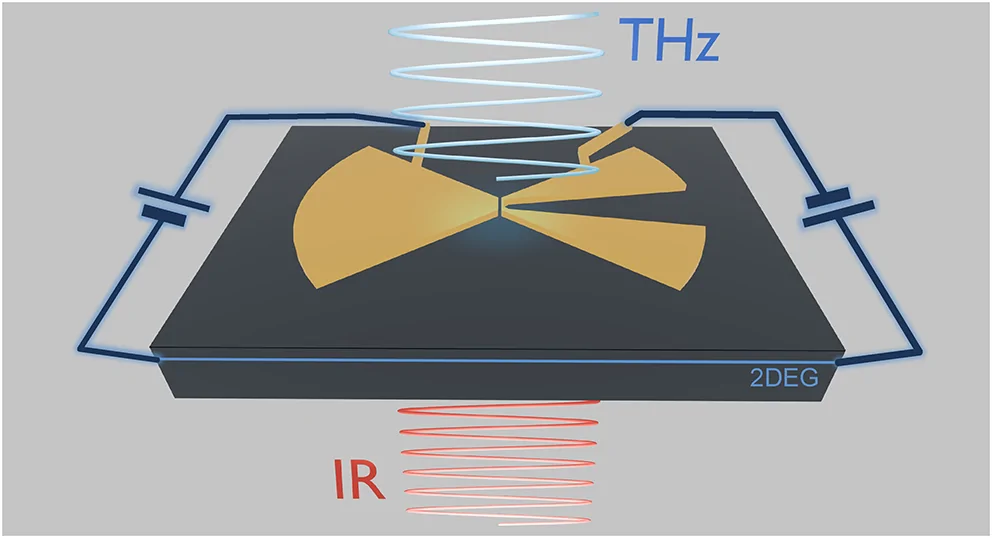

## Introduction

The terahertz (THz) region (*f* = 0.1–10 THz) has received significant attention in recent years due to its unique capabilities in applications such as ultrafast wireless communication,^1,2^ medical imaging^3,4^ and non-destructive testing.^5,6^ The advancement of THz technology critically relies on sensitive detection of terahertz waves. Among the various approaches, devices based on two-dimensional electron gases (2DEGs), such as field effect transistors (FETs), have emerged as a promising platform for THz detection over the past few decades.

The idea of employing FETs for THz detection originated from the proposal of using plasmonic mixing by Dyakonov and Shur in 1996.^7^ Since then, FETs based on a wide range of material systems, including gallium arsenide,^8–10^ silicon,^11–13^ and gallium nitride,^14,15^ have been explored as THz detectors utilizing plasmonic mixing as the underlying mechanism.^16,17^ Other mechanisms responsible for the generation of a THz photoresponse in 2DEG-based THz detectors have also been demonstrated, including photo-thermoelectric effect,^18,19^ collision-less electron heating,^20^ photon-assisted tunneling,^21^ bolometric,^22^ and photovoltaic effects.^23^

In 2022, a novel, quantum, collision-free mechanism, the in-plane photoelectric (IPPE) effect, was demonstrated.^24,25^ Devices exploiting the IPPE effect for THz detection are called photoelectric tunable-step (PETS) THz detectors. The detection mechanism is illustrated in Figure 1: by applying two different voltages onto independently biased gates, the bottoms of the conduction band under the left and right gates shift, respectively, and an electrically tunable potential step is artificially created within the channel. Under terahertz illumination, the antenna on top of the detector couples the incident terahertz radiation into the 2DEG and amplifies the electric field within the gap between the two gates. Electrons near the artificially created potential step absorb photons, which leads to a net electron flow of quantum origin within the 2DEG from the high- to the low-electron-density area. This results in a strong photoresponse of the device generated under zero source-drain bias.

**Figure 1:**
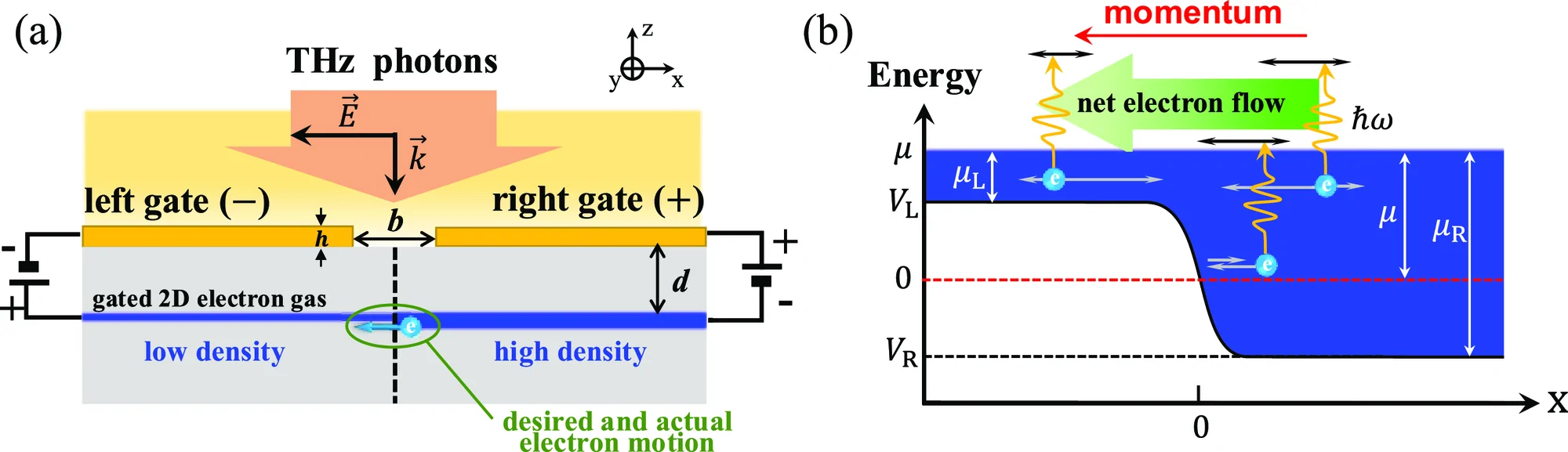
Physical principle of the in-plane photoelectric effect. (a) Side view of a photoelectric tunable-step THz detector. The incident THz radiation is coupled and amplified into the 2D electron channel by the gates on top. (b) An electrically tunable potential step created by biasing the left and right gates differently. The photoexcited electrons move from the high- to the low-electron-density regions, leading to a net electron flow onto the potential step. Different from the conventional photoelectric effect, the momentum of electrons in the in-plane photoelectric effect aligns with the electric field of the incident wave.

The first PETS detectors with a bow-tie antenna on top showed a strong photoresponse to ∼1.9 THz radiation in an AlGaAs/GaAs high electron mobility transistor heterostructure at 9 K.^24^ Subsequent studies further improved the responsivity of PETS THz detectors by optimizing devices’ dimensions and revealed that the IPPE effect in AlGaAs/GaAs heterostructures persists up to 75 K, significantly extending its practical applicability.^26^ Besides single-pixel antenna geometries, including dipole,^26–28^ bow-tie,^24^ and X-wing^29^ antennas, high-sensitivity PETS THz detectors with metasurface structures were recently demonstrated.^30^ The observation of the IPPE effect in an AlGaN/GaN heterostructure shows its universality across different material systems supporting a 2DEG.^31^

A comparison of the performance of PETS THz detectors exploiting the IPPE effect with that of other state-of-the-art THz and sub-THz detectors is presented in Table 1. Superconducting hot-electron bolometers (HEBs) show excellent performance with high responsivity, low noise equivalent power (NEP), and short response time in the THz range.^32,33^ However, expensive cryogenic temperature operation is required since SHEBs are inherently limited by the superconducting transition temperatures (*T*_c_), for example, *T*_c_ = 8.5 K for 10 nm MgB_2_ film,^33^ *T*_c_ = 9.9K for NbN film.^34^ By contrast, PETS THz detectors offer a more cost-effective solution since they are capable of operating at much higher temperatures, up to 75 K for AlGaAs/GaAs-based devices,^26^ with potential to operate at even higher temperatures on other material systems. Photonic detectors based on intersubband or intersublevel transitions, including quantum well infrared photodetectors (QWIPs) and quantum dot detectors (QDDs), exhibit large responsivities and fast response speeds^35–37^ but require an applied source-drain bias and exhibit a rapid decay of the responsivity above liquid helium temperatures. For instance, an InAs/GaAs quantum ring intersublevel detector, formed by postepitaxy thermal annealing of quantum dots, demonstrates a high responsivity of 25 A/W under 1.82 THz radiation at 5.2 K, which drops significantly to 2.1 A/W at 10 K, with no detectable signal observed at 12 K.^35^ On the contrary, PETS THz detectors operate without source-drain bias, and the decay of responsivity with increasing temperature is much smaller.^26^

**Table 1:** Comparison of Photoelectric Tunable-Step (PETS) THz Detectors Based on the In-Plane Photoelectric (IPPE) Effect with Other State-of-the-Art THz and Sub-THz Photodetectors^a^

detector	mechanism	*f* (THz)	*T* (K)	$R_{I}$ (mA/W)	$R_{V}$ (V/W)	NEP $\left(\mathrm{pW}/\sqrt{\mathrm{Hz}}\right)$	τ (μs)
Terahertz Detectors							
bowtie PETS^24^	IPPE	1.9	9	77	1.4 × 10^3^		2
dipole PETS^26^	IPPE	1.9	8	128	2.5 × 10^3^		2
MetaPETS^30^	IPPE	1.9	10	2.7 × 10^3^		0.39	2
MoRe HEB^32^	HEB	0.3–3	4.2		10^4^	0.05–0.07	10^–3^
MgB_2_ HEB^33^	HEB	1.63	4.2		1–2 × 10^3^		
QWIP^36^	ISTs	3	4.5	8 × 10^2^			5 × 10^–5^
QWIP^37^	ISTs	5.2	25	250		0.2 (15 K)	
QRID^35^	ISTs	1.82	5.2	25 × 10^3^			3 × 10^–4^
			10	2.1 × 10^3^			
			12	no signal			
Si CMOS FET^38^	plasmonic	2.52	room			63	
Si CMOS FET^39^	plasmonic	3.1	room		230	85	
Si CMOS FET^17^	plasmonic	2.9	room		30	478	
bilayer graphene FET^40^	plasmonic	2	10		240		
graphene FET^41^	plasmonic	4.7	10	1.5 × 10^–3^			
graphene FET^23^	photovoltaic	2	room	3.4 × 10^–3^		1.5 × 10^5^	
graphene FET^42^	PTE	2.52	room		0.364	1.3 × 10^4^	376
graphene FET^43^	PTE	3.4	room		50	120	8.9 × 10^–4^
graphene pn junction^44^	PTE	2.52	room	25	105	80	0.03
Sub-Terahertz Detectors							
hBN/BP/hBN FET^45^	plasmonic, bolometric, PTE	0.295	4		38	100	
p-n graphene grid^46^	bolometric	0.177	<1	1		200	6 × 10^–3^
quantum point contact^20^	collisionless e-heating	0.11–0.17	4.2		10^6^		
graphene FET^47^	PTE	0.3	4	55.6		1.3 × 10^3^	
			room	1.28		9 × 10^5^	

a HEB, hot electron bolometer; QWIPs, quantum well infrared photodetectors; ISTs, intersubband/intersublevel transitions; QRIDs, quantum ring intersublevel detectors; and PTE, photothermoelectric effect.

Various FET-based detectors relying on alternative detection mechanisms have been reported, e.g., silicon-based FETs utilizing plasmonic mixing, graphene-based devices exploiting the photothermoelectric effect, and others.^20,23,40–47^ Among detectors based on 2D electron systems, the novel PETS THz detectors are highly promising devices, as the in-plane photoelectric effect underpinning their operation was shown to provide a substantially larger response^24^ than other, previously known mechanisms, including plasmonic mixing and photothermoelectric effects. Since their discovery in 2022, the field of PETS detectors has been rapidly developing.^26^ Nowadays, experimentally achieved responsivity values surpass the performance of other detectors at comparable frequencies and operating temperatures.^30^ For example, a dipole-antenna PETS THz detector achieved a photovoltage responsivity of 2.5 kV/W to ∼2 THz radiation at 10 K,^26^ which is notably higher than the 240 V/W responsivity of a bilayer-graphene FET under comparable conditions, that relied on plasmonic mixing.^40^ More recently, a quantum metasurface-based PETS detector achieved a responsivity of 2.7 A/W at 10 K, exceeding that of single-pixel devices by more than 20 times.^30^ Although some devices exhibit comparable or even higher responsivities, they operate at significantly lower frequencies; for example, a responsivity of 10^6^ V/W was reported in an AlGaAs/GaAs-based quantum point contact operating at 0.11–0.17 THz based on collision-less electron heating.^20^ Achieving high-sensitivity detection with FET-based THz detectors above 1 THz remains challenging, as the responsivity drops and the noise-equivalent power rises with frequency.^17,38^ For broadband detectors, the decay was found to be approximately ω^–3^ with frequency for antenna-coupled FETs^48,49^ and, in some cases, an even steeper ω^–7^ dependence^50^ was observed. This further highlights the highly promising performance of PETS THz detectors based on the IPPE effect, which is predicted to operate across the entire THz range.^24,25^

Despite the outstanding performance and rapid development of detectors based on the IPPE effect, a fundamental question remains unresolved: how does the performance of PETS THz detectors depend on the electron density in the 2DEG channel? In AlGaAs/GaAs heterostructures, the electron density can be tuned in situ via the persistent photoconductivity (PPC) effect, a unique phenomenon associated with the photoionization of DX centers, in which optical illumination under low-temperature conditions (typically *T* < 100 K) induces a long-lived increase in electron density that persists for extended periods ranging from hours to days.^51–54^ The PPC effect provides a convenient and continuous method of modulating the electron concentration in 2DEGs over a wide range, as demonstrated in refs 55,56 .

In this work, we systematically investigate the dependence of the IPPE effect on the electron density in the 2DEG by exploiting the PPC effect in an AlGaAs/GaAs-based PETS THz detector. Incremental infrared illumination with small doses enables the controlled tuning of electron density and mobility in the 2DEG channel. Our results reveal the existence of an optimal illumination dose that maximizes detector performance, providing a practical strategy for the performance optimization of PETS THz detectors through infrared illumination. This study also elucidates the underlying mechanisms by which infrared illumination modulates the IPPE effect in AlGaAs/GaAs-based two-dimensional electron systems.

## Device Design

A bowtie-antenna PETS THz detector as shown in Figure 2a,b is fabricated from an AlGaAs/GaAs high electron mobility heterostucture. The layer structure will be discussed later in detail, in Figure 6c. The 2DEG is approximately 77 nm beneath the top surface, which is estimated from the electron density vs gate voltage data shown in Supporting Information Section 1. The 2DEG is characterized by Hall measurements and features an electron density of 2.3 × 10^11^ cm^–2^ and a mobility of 5.4 × 10^5^ cm^2^/V s in the dark at 1.5 K.

**Figure 2:**
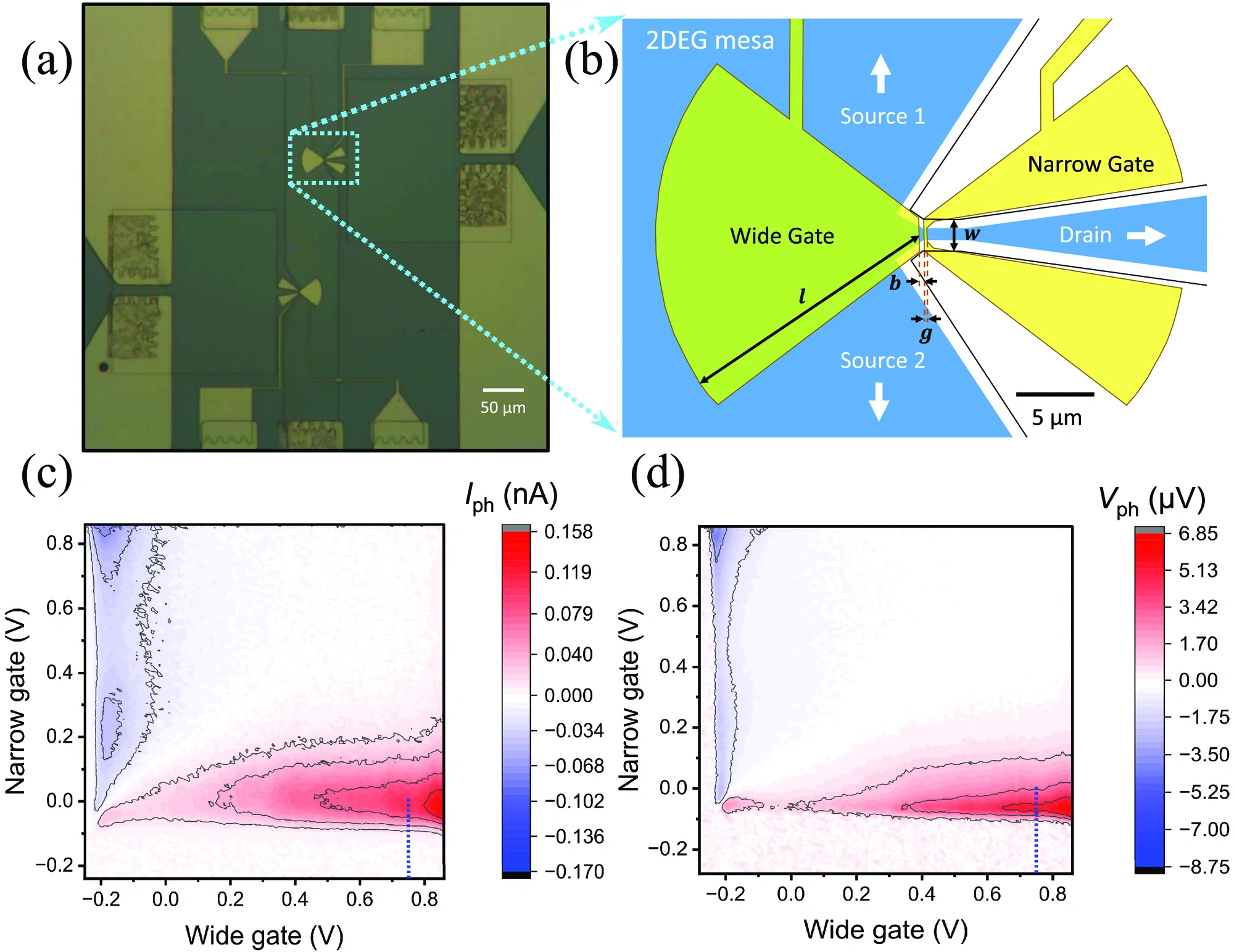
Photograph and characterization data of the AlGaAs/GaAs-based PETS THz detector. (a) Optical microscope image of the device. (b) Schematic diagram of the bow-tie antenna at the top of the detector. The dimensions of the bow-tie antenna are radius *l* = 17 μm, gap size *b* = 270 nm, mesa width *w* = 2 μm, and bridge width of the narrow gate *g* = 200 nm. (c, d) Photoresponse characterization of the PETS THz detector in the dark after cooldown to 6 K, with (c) photocurrent and (d) photovoltage. The vertical dashed blue lines at 0.75 V wide gate voltage indicate the gate biases along which a line scan is studied in dependence on infrared illumination dose in the subsequent Figures 3a,b and 4a.

The PETS THz detector represents a dual-gated antenna-coupled FET with the two gates shaped in the form of a bowtie antenna. The antenna confines incident radiation into a narrow channel, created within the heterostructure by mesa etching. The two Ti/Au wings of the bowtie antenna serve as the two gates of the detector, with one gate being solid, while the other gate having a cutout that narrows the metal down to a narrow bridge at the antenna center. Annealed AuGeNi ohmic contacts far away from the active region serve as sources and drains for electron transport in the 2DEG. The dimensions of the bowtie antenna on top of the device are identical to those in ref 24.

## Experimental Setup

The PETS THz detector is placed in a cryostat, which enables it to be excited by THz radiation from the top (antenna side) using a quantum cascade laser (QCL) and illuminated with an infrared LED from the backside, through the 500 μm GaAs semi-insulating substrate. The QCL used^57^ emits at a wavelength of λ = 159.38 μm (1.881 THz emission frequency).

The detector and the QCL are cooled down to ∼6 and ∼18 K, respectively, in two liquid helium continuous-flow cryostats. A cylindrical multimode waveguide system (annealed copper waveguides, 4.6 mm inner diameter) delivers and focuses the THz radiation from the QCL to the front surface of the detector.^58^ The QCL is electrically modulated by a square wave with a frequency of 772 Hz and a duty cycle of 2.14 %, enabling measurements of the photoresponse using a lock-in technique.

The infrared (IR) illumination is realized using an LED operated at room temperature with an IR center wavelength of λ = 940 nm (*E* = 1.32 eV). A 1 mm diameter hole throughout the copper support of the sample enables unobstructed access for illumination of the detector back side; see Supporting Information Section 2. The substrate-side illumination ensures homogeneous excitation of the 2D electron system, preventing a shadowing effect from the gates that would otherwise occur in the case of top-side IR illumination.

## Results

After the PETS detector is cooled down, its photocurrent and photovoltage characteristics are recorded in the dark, yielding Figure 2c,d. A response that is typical for PETS detectors is observed,^24,26^ with a response above the gate thresholds and a polarity reversal of the photocurrent/-voltage as the polarity of the gate voltages is flipped.

To investigate the influence of infrared illumination on the AlGaAs/GaAs-based PETS THz detector performance, it is exposed to a sequence of flashes by the LED, with the detector performance recorded in the dark, at the beginning, and after each pulse. Before exposing the PETS detector to a flash of IR light, all gate voltages are tuned to 0 V. After each flash, at least 5 minutes are given for the device to stabilize. Thereafter, the gate voltages are restored to the operating point (blue dashed line in Figure 2c,d). Biased at 1 mA, the LED emits approximately 20.24 μW of 940 nm infrared radiation through a 1 mm diameter hole on the copper holder toward the back of the detector, as discussed in Supporting Information Section 2. A 1-second illumination at this power yields an energy of 20.24 μJ, which serves as the unit illumination dose in the experiment. The term “illumination time” used below refers to the total accumulated dose of infrared illumination, normalized with respect to a unit energy dose of 20.24 μJ.

To measure the photocurrent and photovoltage responses of the PETS THz detector, a fixed bias of 0.75 V was applied to the wide gate of the device, while the narrow gate was scanned from −0.3 to 0 V to investigate the optimal photoresponse. As illustrated in Figure 3a,c, the maximum photocurrent response, max­(*I*_ph_), shows an initial decrease until ∼10 s, and then rises sharply with continued illumination. Following saturation around 10^5^ s, further illumination leads to a decrease in max­(*I*_ph_).

**Figure 3:**
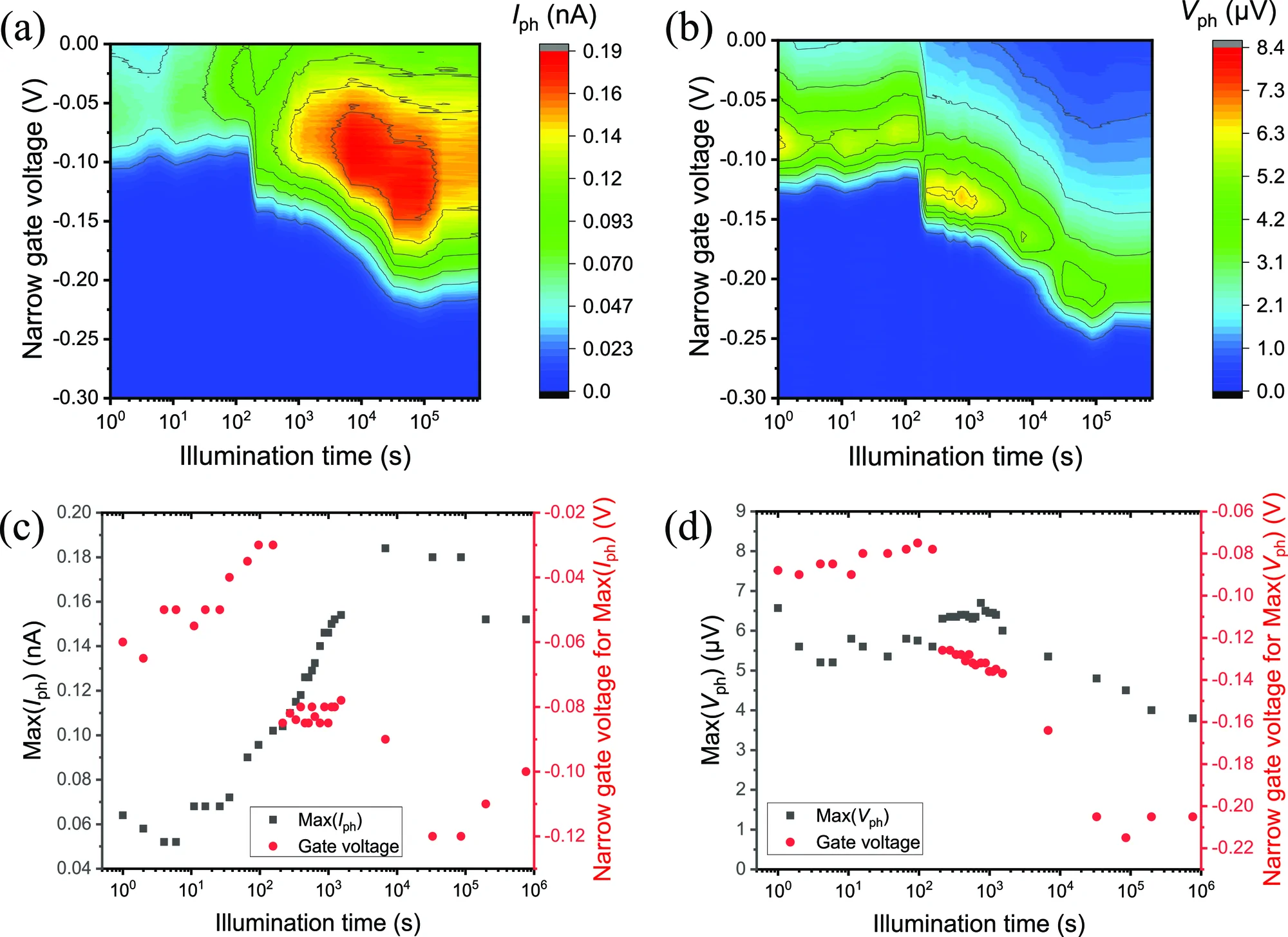
Dependence of the photoresponse of the AlGaAs/GaAs-based PETS THz detector on infrared illumination dose. (a) Photocurrent and (b) photovoltage responses as functions of the narrow gate voltage and the illumination time. (c) The maximum photocurrent response (black squares) and the corresponding narrow gate voltage (red dots) as functions of the illumination time. (d) The optimal photovoltage response (black squares) and the corresponding gate bias (red dots) as functions of the illumination time. The graphs are plotted as a function of the total accumulated duration of IR illumination, normalized to the equivalent illumination dose of 20.24 μJ. The wide gate of the PETS THz detector is biased with 0.75 V. The operating temperature of the device is around 6 K.

The narrow gate voltage that yields max­(*I*_ph_) also shifts with illumination. As shown by the red dots in Figure 3c, the gate voltage operating point initially increases at the beginning, and then a sudden drop from −0.03 to −0.085 V happens around 200 s. With continued illumination, the optimal gate bias drops gradually and then rises up slightly after saturation. Compared with the significant change in photocurrent, the change in the photovoltage is limited, as demonstrated in Figure 3b,d. The maximum photovoltage response, max­(*V*_ph_), is stable until ∼1500 s and then drops down gradually after saturation. The shift of the narrow gate voltage for max­(*V*_ph_) is similar to that for max­(*I*_ph_) in Figure 3c, including the sudden change at around 200 s.

The increase of the photoresponse is consistent with the enhancement of the conductance of the 2DEG channel, illustrated in Figure 4. The conductance, measured in a two-terminal configuration, increases significantly with infrared illumination, and the threshold voltage shifts towards negative values. Comparing the conductance of the 2DEG after full illumination (∼10^6^ s, at which saturation is achieved) with that before illumination, as depicted in Figure 4b, shows that the conductance at *U*_G,narrow_ = 0 is approximately 14 times higher and the threshold voltage *U*_th_ shifts from ∼−0.1 to ∼−0.2 V. This is consistent with the persistent photoconductivity effect (PPC) in AlGaAs/GaAs structures, and results in an increase of both the conductance and photoresponse in PETS THz detectors.

**Figure 4:**
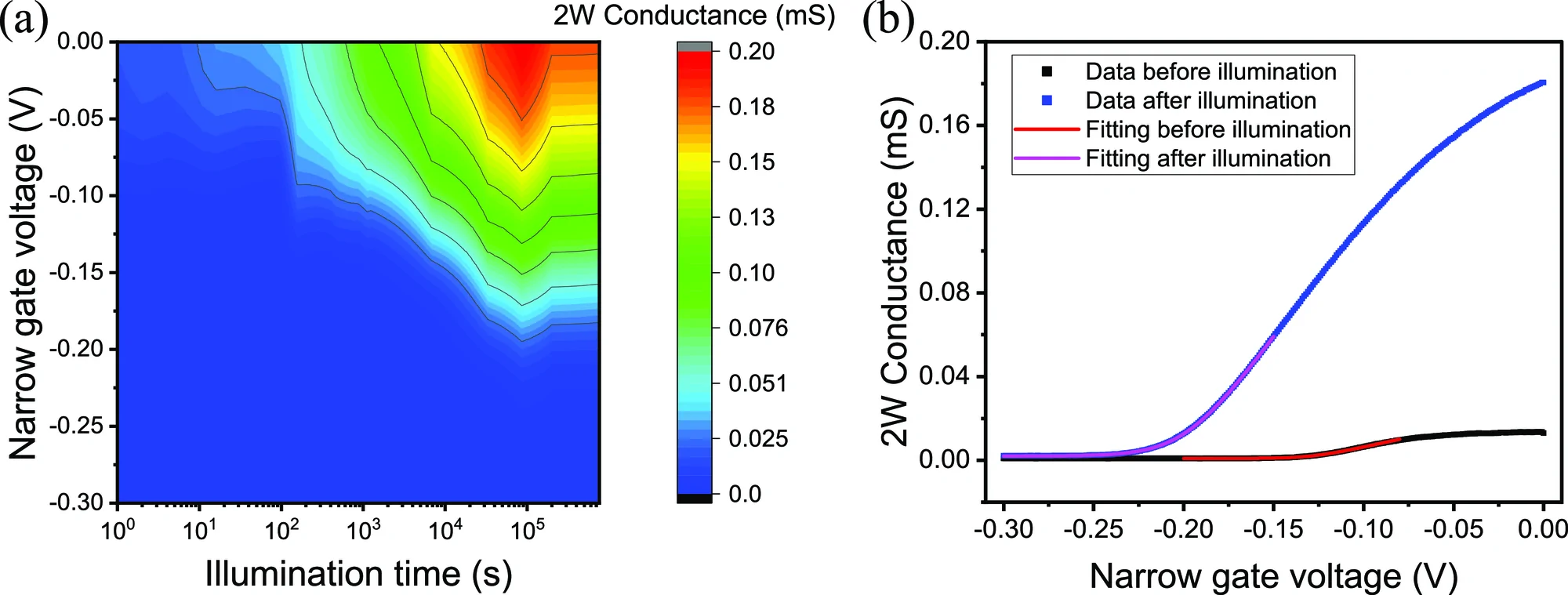
Dependence of the conductance of the PETS THz detector on infrared illumination dose. (a) Two-terminal conductance as a function of the narrow gate voltage and the illumination time. (b) Two-terminal conductance as a function of the narrow gate voltage before infrared illumination and after saturation under infrared illumination. The experimental data are fitted using eq 2 for the threshold voltage *U*_th_.

In the bowtie-antenna PETS THz detector, metal gates are evaporated onto a wafer with a 2DEG 77.4 nm beneath the top surface, enabling estimation of the equilibrium charge carrier density *n*_0_, i.e., the charge carrier density at zero gate voltage *n*(*U*_G_ = 0), using the parallel-plate capacitor model

1 $$n_{0}=n\left(U_{G}=0\right)=-\frac{CU_{\mathrm{th}}}{\mathrm{eA}}=-\frac{\varepsilon_{0}\varepsilon_{r}U_{\mathrm{th}}}{\mathrm{de}}$$

where ε_r_ is the relative dielectric constant for GaAs and *d* is the distance from the surface gates to the 2DEG. The shift of the threshold voltage *U*_th_ results in a change of the equilibrium charge carrier density *n*_0_.

To obtain an accurate value of the threshold voltage *U*_th_, the conductance around the threshold region is fitted as a function of the narrow gate voltage; see Figure 4b

2 $$f\left(U_{G}\right)=A\ln\left(1+\exp\left(\frac{e\left(U_{G}-U_{\mathrm{th}}\right)}{\eta k_{B}T}\right)\right)$$

where *k*_B_ is the Boltzmann constant, *T* is the absolute temperature in Kelvin, and η is the dimensionless ideality factor. The threshold voltage *U*_th_ after a sequence of flashes is demonstrated in Figure 5a (black squares), and the corresponding equilibrium electron density *n*_0_ calculated from eq 1 is presented in Figure 5a with red dots.

**Figure 5:**
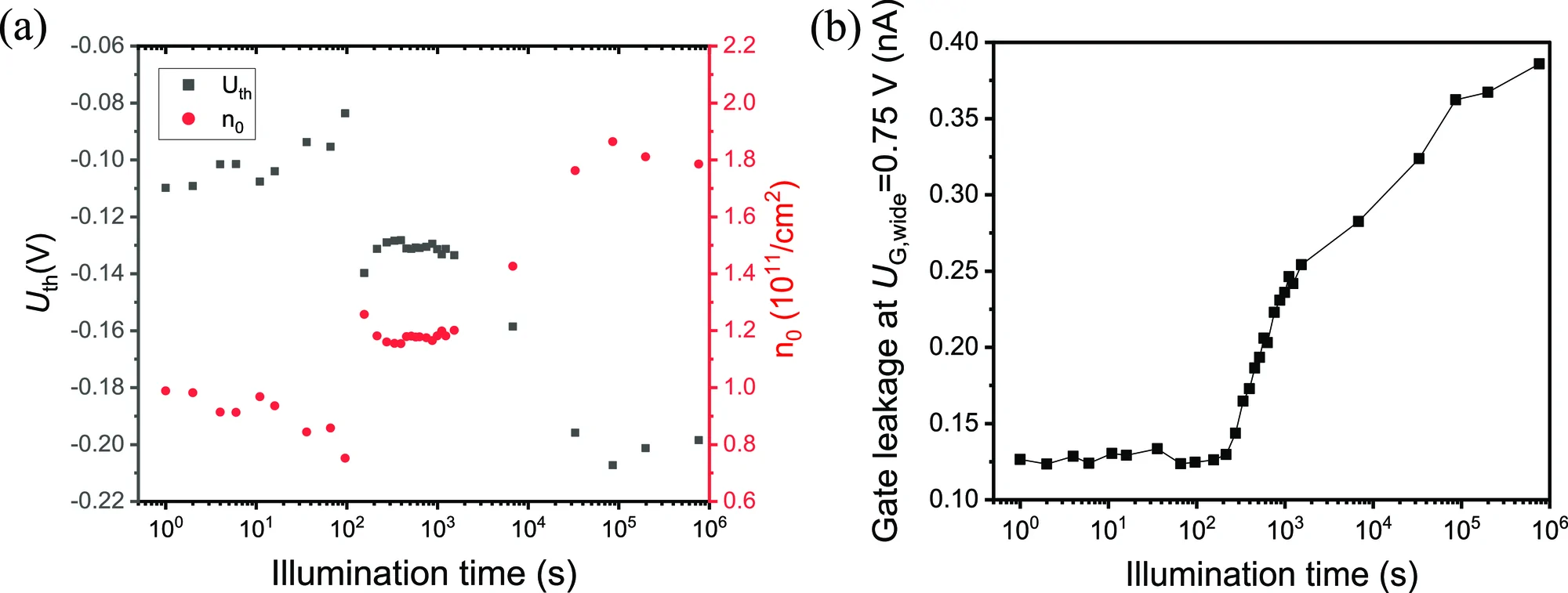
Influence of infrared illumination on electron density and gate leakage. (a) The threshold voltage *U*_th_ (black squares) and the corresponding equilibrium charge carrier density *n*_0_ (red circles). The threshold voltages *U*_th_ are obtained by fitting the 2-wire conductance curves using eq 2. The equilibrium charge carrier densities *n*_0_ are calculated from *U*_th_ using eq 1. (b) Leakage current of the wide gate of the PETS THz detector in dependence on infrared illumination dose, with the wide gate biased at 0.75 V.

In the dark, the PETS THz detector features an equilibrium charge carrier density of ∼1 × 10^11^ cm^–2^. Once infrared illumination starts, *n*_0_ decreases slightly, and then at around 100 s, *n*_0_ rises up steeply to a plateau with *n*_0_ = 1.2 × 10^11^ cm^–2^. Beyond 1500 s, *n*_0_ gradually increases until it saturates at approximately 1.8 × 10^11^ cm^–2^. The increase in charge carrier density within the 2DEG channel after full infrared illumination is consistent with the enhancement of the photoresponse (Figure 3) and the conductance (Figure 4).

During all measurements, the wide gate voltage was set to 0.75 V. As shown in Figure 5b, the gate leakage at 0.75 V initially stays constant but then gradually increases after 200 s. However, the gate leakage is negligible during the entire illumination, with 0.4 nA being the highest value recorded.

The reproducibility of the behaviors reported above was confirmed through multiple independent cooldown and measurement cycles, which yielded consistent results. A representative example is shown in Supporting Information Section 3.

## Interpretation

The change in carrier density after illumination is mostly attributed to the existence of deep-level donor impurities in semiconductors. These deep levels are known as DX centers,^59–64^ which are complexes involving a donor atom (D) and an anion vacancy (X), e.g., Si–V_As_ for Si-doped Al_*x*_Ga_1–*x*_As.^60,65^ The most successful model to explain the DX center is the “negative-U model” proposed by Chadi and Chang,^62,66^ supported by ref 53, and further analyzed in ref 67. The DX center is a highly localized and negatively charged center resulting from the reaction

3 $$2d^{0}\to d^{+}+{\mathrm{DX}}^{-}$$

where d^0^ and d^+^ represent neutral and ionized four-fold-coordinated substitutional donors. The density of DX centers is, at most, one half the density of the donor atoms. In highly doped samples, DX centers may be formed by capturing two electrons^66^

4 $$d^{+}+2e^{-}\to {\mathrm{DX}}^{-}$$

The ionization and capture processes of DX centers go through two steps

5 $${\mathrm{DX}}^{-}⇆d^{0}+e^{-}⇆d^{+}+2e^{-}$$

where the intermediate neutral d^0^ state is thermodynamically unstable but plays a role in all carrier capture and emission processes of DX centers.^67^ An important property of DX centers is that, once ionized, their electron capture rate is thermally activated and strongly suppressed at liquid helium temperatures. This gives rise to a persistent photoconductivity effect: once the electron density is changed due to DX center ionization, it remains in the non-equilibrium state for a long time (hours to days).^51–56^

As illustrated in Figure 5a, the equilibrium charge carrier density *n*_0_ in the 2DEG decreases slightly at the beginning before rising up steeply to a plateau at around 200 s, and then stays at the plateau until ∼1500 s. Beyond 1500 s, *n*_0_ gradually increases until it saturates. The overall increase in *n*_0_ is attributed to the ionization of DX centers, from which electrons are released and tunnel through into the 2DEG channel, resulting in an overall rise in electron density. However, the presence of a plateau between ∼200 and ∼1500 s, as well as the two-stage growth behavior of *n*_0_, suggests that additional mechanisms may be involved that require further explanation.

First, we exclude parallel conduction through the doped layer as a possible cause of the observed behavior. In AlGaAs/GaAs high-electron-mobility transistor heterostructures such as the one we use here, see Figure 6c, such a phenomenon could occur and may lead to abrupt levelling-off of the electron density^53^ in the 2DEG, similar to what is observed here at ∼200 s. However, magnetotransport measurements on a reference Hall bar from the same wafer show that no parallel conduction is present, even under high gate bias and after red-light illumination. Only one 2DEG channel around 77 nm below the top surface is observed; see Supporting Information Section 1.

**Figure 6:**
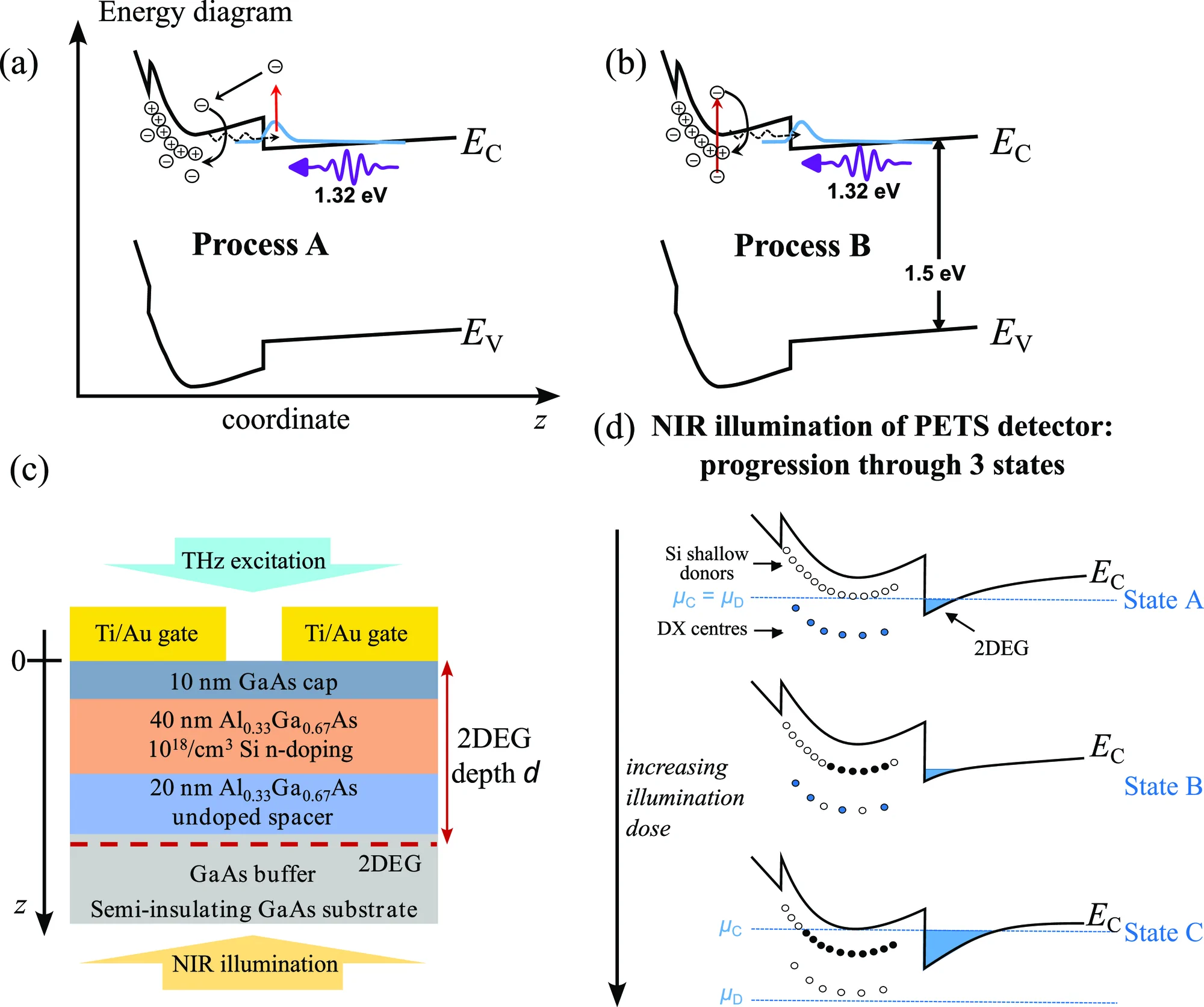
The AlGaAs/GaAs heterostructure and processes occurring during infrared illumination. (a, b) Schematic diagrams of two processes occurring under infrared illumination. Process A: excitation of electrons from the 2DEG, with relaxation into the doped barrier region. Process B: DX-center excitation within the barrier region. The bandgap energies of GaAs and Al_*x*_Ga_1–*x*_As (where *x* ∼ 0.3) are 1.5 and 1.9 eV, respectively, larger than the photon energy, 1.32 eV, from the infrared LED. This figure is adapted from ref 68 with modifications. (c) The layers of the AlGaAs/GaAs heterostructure employed in the PETS detector under study. The 2DEG is located approximately *d* = 77 nm below the top surface. (d) Conduction band diagram of the PETS detectors undergoing three different stages upon infrared illumination. Si shallow donors and DX centers are represented by circles: white-filled for positively charged, black-filled for neutral, and blue-filled for negatively charged states. μ_c_ represents the quasi-Fermi level for the conducting electrons and Si shallow donors, and μ_d_ refers to the quasi-Fermi level for the deep DX centers. Before illumination, the semiconductor heterostructure can be characterized by a single Fermi level with μ_c_ = μ_d_. However, after illumination, μ_c_ and μ_d_ drift apart.

We now present an explanation of the observed behavior based on DX-center excitation in combination with a backfilling of Si donor levels that occur through three stages as the illumination dose is increased, which is shown in Figure 6d.

For an AlGaAs/GaAs heterostructure, typical observed thresholds of photon energies for changing the photoconductivity of a 2DEG are 1.9 eV corresponding to the bandgap of Al_*x*_Ga_1–*x*_As (where *x* ∼ 0.3), 1.5 eV corresponding to the bandgap of GaAs, 1.1 eV corresponding to DX center activation, and 0.6-0.8 eV corresponding to other traps, very likely in the GaAs buffer layer.^68–71^ Here, we illuminate the sample with a photon energy of 1.32 eV, which is smaller than the GaAs and Al_*x*_Ga_1–*x*_As bandgap energies. In this case, two photoexcitation processes, called A and B can occur, as shown in Figure 6a,b. Process A corresponds to the excitation of electrons from the 2DEG, with relaxation into the doped barrier region. Process B corresponds to DX-center excitation within the barrier region.

After cooling in the dark and before illumination, the sample is in thermal equilibrium. The DX centers are not ionized, while part of the Si donors are ionized and contribute to the creation of the electrons in the 2DEG (State A in Figure 6d). As the sample starts to be illuminated, both processes A and B will occur simultaneously.

In process A, electrons are photoexcited, and these photoexcited electrons will experience the electric field acting on them due to the tilt of the conduction band edge. Those photoexcited electrons that go lower into the heterostructure (to the right in the figure) will relax and return to the 2D electron gas. Those electrons that go to the left in the figure will accumulate in the potential minimum created by the ionized silicon donors, which are positively charged. While the electrons will not be captured again by the DX centers, whose capture rate is thermally activated and suppressed at 6 K, they will compensate some of the ionized Si shallow donors. Simultaneously, the near-infrared LED will ionize DX centers, as shown by process B, thus creating electrons in the conduction band of the doped region. Some of these electrons will relax into the conduction-band minimum of the doped donor region. Other electrons relax into the triangular quantum well.

At low illumination doses (<100 s), the threshold voltage initially shifts to more positive values corresponding to a reduction of the electron density in the channel (State A to B in Figure 6d). At this stage, process A is dominant since most electrons photoexcited from the DX centers will be captured by the ionized Si shallow donors.^51^ Therefore, after a small amount of photoexcitation, this will reduce the electron density in the 2DEG (Figure 5a). However, the scattering is reduced, as the ionized Si shallow donors are neutralized after illumination. The lower electron density results in an overall reduction in remote impurity scattering and leads to increased mobility.

From ∼200 s, the ionization of the DX centers, process B, becomes the dominant process. More electrons are released from the DX centers. Some of them tunnel through the barrier into the 2DEG, which is compensated by the decrease of electrons by process A, hence keeping the electron density in the channel constant over a certain range of illumination doses (from ∼200 to ∼1500 s). However, the plateau does not mean that the system is stationary, because with every additional illumination pulse, more DX centers become ionized in the doped region, and the quasi-Fermi levels for electrons and holes start to drift apart more and more. Hence, in the doped region, there are more and more positively charged DX centers, and simultaneously there are more electrons that are filling back the ionized silicon donors. At some point, most of the ionized silicon donors contributing to the 2DEG will be filled back, and the electrons will start to fill states in the conduction band edge of the parabolic minimum. Soon after that, however, they will start to tunnel through into the 2DEG, now notably increasing the 2D electron gas density, thanks to the large number of ionized DX centers, which manifests itself in the threshold voltage shifting back again to more negative values.

As the illumination dose keeps increasing, more and more impurities/DX centers become ionized in the doped region, and the contributing electrons accumulate slowly in the 2DEG from ∼1500 s until saturation ∼10^5^ s (State B to C in Figure 6d). The larger amount of positively charged impurities in the doped region now pulls the conduction band edge down in the region from 10 to 50 nm, resulting in a steeper and thinner triangular potential barrier at the surface facing the gate metal. This reduces the size of the tunneling barrier from the surface to the conduction band minimum formed in the doped region, resulting in a higher electron tunneling rate. As a result of that, the gate leakage starts to occur at lower gate voltage and keeps increasing from that point, as seen in Figure 5b.

Attention is now directed to the magnitude of the observed photoresponse in Figure 3.

The strength of the in-plane photoelectric current depends physically on the chemical potentials under the positively and negatively biased gates,^24^ and experimentally also on the mobility of the electrons in the channel. Illumination of the sample with the infrared LED hardly affects the conditions of the 2DEG under a strongly positively biased gate. Under such high positive gate voltages, when the 2D electron gas operates in enhancement mode, it is known that the density of electrons does not depend anymore on whether the sample has been illuminated or not.^72^ Moreover, as under the positively biased gate the electron density is already very high, any change of the electron density induced by additional donor ionization will not significantly alter the parameters of the 2DEG under that gate. The highest photoresponse is observed close to the threshold of the device, and correspondingly, the position of the photoresponse shifts together with the threshold voltage of the device. The main reason for the change in the photoresponse magnitude is related to the scattering and, hence, the mobility of electrons under the negatively biased gate.

At low illumination doses (<100 s), the electron density decreases, but the photoresponse stays the same or slightly increases. The backfilling of the ionized silicon donors reduces the scattering in the channel, hence enhancing the mobility under the negatively biased gate, which in turn results in a slight increase of the observed photoresponse, despite a reduction of the density/absolute value of the threshold voltage.

At the region from around 200 to 1500 seconds, just enough DX centers are ionized to create the optimal regime of photovoltage detection. Electrons excited from the DX centers compensate the ionized Si shallow donors, reducing the scattering and improving the mobility. Therefore, the photovoltage response increases to the optimal value, as any electrons photoexcited by the THz radiation experience the least amount of scattering.

The maximum photocurrent response occurs at larger illumination doses around 10^5^ s, while the photovoltage actually decreases in this region. This is because the photocurrent is affected much stronger by changes in the resistance of the channel than the photovoltage. As presented in Figure 4, the significant increase in conductance until ∼10^5^ s due to the illumination of the LED continues to increase the photocurrent, even when the photovoltage starts to decrease.

Finally, after around 10^5^ s of equivalent illumination time, any further increase in illumination causes only a decrease in both *I*_ph_ and *V*_ph_. Here, too many DX centers or deep impurities become ionized, and together with the increasing gate leakage, there may also be accumulation of mobile charges within the doped donor layer that create hopping conduction. Any charge accumulation within the donor layer will screen the incident THz radiation, and if there is any onset of parallel conduction, this will also reduce the IPPE effect by essentially short-circuiting the 2DEG as a detection element. The increasing number of ionized DX centers (i.e., charged remote impurities) will also reduce the mobility and increase gate leakage in the device. Correspondingly, beyond 10^5^ s, both photovoltage and photocurrent responses start to decay.

It should be noted that besides the above-described mechanisms, there is also some charge transfer into the gate metal. As the 2DEG and gates are galvanically connected through the gate circuits, a change in the electron density in the 2DEG also leads to a change in charge on the gate metal. However, this effect has little impact on the band structure: due to the very large density of states in the gate metal, the Fermi level within the gate and at its interface with the semiconductor remains essentially unchanged upon near-infrared illumination. The device characteristics are determined by the band structure changes within the semiconductor, which has a much smaller density of states.

## Discussion and Conclusion

We have shown that infrared illumination is an effective method to dynamically tune the responsivity and output impedance of PETS THz detectors over approximately one order of magnitude. We explained the results using the physics of the persistent photoconductivity effect and the interplay of different donor types. We found that there is an optimal illumination dose that gives the highest terahertz sensitivity and that dose is different depending on whether the detector operates in photocurrent or photovoltage mode. The IR illumination method provides an additional degree of freedom for tuning PETS detector performance that cannot be achieved solely by tuning the two gate voltages and facilitates matching of the device to external readout circuits.

The demonstrated mechanism is highly valuable for scaling up AlGaAs/GaAs-based detector technology to focal plane arrays. In detector arrays, it is very important to minimize pixel-to-pixel deviation of sensitivity in order to produce a reliable 1D or 2D image, e.g., in a cooled high-sensitivity terahertz camera chip—an issue that microbolometer cameras still suffer from. While the sensitivity of a single PETS detector can be tuned using gate voltages, this approach is not suitable for scaling up, as individual fine-tuning of the gate voltages on each pixel is either not possible or very complicated. Our approach provides an elegant solution to this problem: a 2D array of AlGaAs/GaAs-based detectors can be mounted on top of a plate of infrared micro-LEDs, allowing the same gate voltages to be applied to all detectors within the array, with controlled infrared pulses ensuring that the sensitivity of all pixels is set equal. Such an approach will enable in situ calibration of AlGaAs/GaAs-based camera chips, while also mitigating against the performance of individual pixel parameters drifting apart with time.

One challenge in the practical implementation of this tuning mechanism is the persistent nature of DX center ionization at cryogenic temperatures. This leads to a risk of overdosing the device with IR illumination if the detector is illuminated accidentally above the optimal dose. To reset ionized DX centers of the device, a thermal cycling process would be needed. This challenge can be practically solved by placing calibration devices on the same chip away from the active THz detection area/focal plane array, allowing the density vs illumination dose curves to be measured in situ after cooldown using pulsed IR illumination. This allows the key characteristics to be identified, i.e., the illumination dose that results in the sudden threshold voltage shift and the illumination dose where the density stops increasing. This calibration can be done on off-camera calibration units but on the same substrate. Thereafter, the dose to be applied to the infrared LEDs backing the THz detector array is known. In addition, instead of aiming to reach the dose yielding maximum responsivity, in practical operation, it is sensible to aim for a slightly lower infrared illumination dose in order to avoid accidental overdosing. A drift of the electron density will reduce the electron density with time, as electrons are recaptured by ionized DX centers, a process that is slow at cryogenic temperatures but still occurs on time scales of hours to days. At periodic intervals, an in situ recalibration of the camera can be done, where small additional pulses of infrared radiation are applied to those pixels whose sensitivity or density appears to have drifted away to restore uniform responsivity across the focal plane array. Such periodic internal recalibrations are common in, e.g., modern oscilloscopes. These issues can be solved in practical device implementations via device firmware and internal calibration lookup tables.

The demonstrated method is a useful tool for in situ tuning of all optoelectronics relying on AlGaAs/GaAs heterostructures. This performance optimization method can be exploited to facilitate scalable terahertz optoelectronic devices and arrays that utilize physical concepts from both the infrared and terahertz spectral ranges.

## Supplementary Material



## Notes

Longitudinal resistivity and transverse resistivity of the 2DEG in the AlGaAs/GaAs wafer; electron density and mobility of the 2DEG as functions of the gate voltage; setup for the back-side infrared illumination; photos and LIV curves of the infrared LED; reproducibility measurements from an independent cool-down of the PETS THz detector under IR illumination (PDF)
