# Supplementary material for: Infrared Illumination-Induced Responsivity Enhancement in Photoelectric Tunable-Step Terahertz Detectors
Source: ACS Photonics. 2026 Sep 4;13(19):5749–60. doi: 10.1021/acsphotonics.6c01501 (PMC13651708; doi:10.1021/acsphotonics.6c01501)
Supplement: Supplementary file 1 [file ph6c01501_si_001.pdf]

Supplementary information to: Infrared illumination-induced responsivity enhancement in photoelectric tuneable-step terahertz detectors

Ran Chen*[1,2], Matthew Tan[1], Harvey E. Beere[1], Jonathan P. Griffiths[1], David A. Ritchie[1,3,4], Wladislaw Michailow*[1,3,4]

[1]Cavendish Laboratory, University of Cambridge, CB3 0HE Cambridge, UK
[2]Center of Ionic Liquids and Green Energy, Beijing Key Laboratory of Solid State Battery and Energy Storage Process, Institute of Process Engineering, Chinese Academy of Sciences, Beijing 100190, China
[3]Centre for Integrative Semiconductor Materials, Swansea University, SA1 8EN Swansea, UK
[4]Department of Physics, Swansea University, SA1 8EN Swansea, UK

*chenran@ipe.ac.cn (R. Chen), wladislaw.michailow@swansea.ac.uk (W. Michailow)

1 Magnetotransport measurements

Standard gated Hall bars were fabricated from the same wafer shown in Fig. 6(c), with a 12 nm-thick NiCr gate deposited on top of the Hall bar serving as a semi-transparent gate. While the Schottky barrier height of NiCr (0.77 eV for both Ni and Cr) is slightly different from that of Ti/Au, the material used for the gates in the PETS detectors, (0.83 eV for Ti and 0.89 eV for Au),[S1] the NiCr-gated Hall bar remains adequate for evaluating whether parallel conduction occurs in the wafer.

The gated Hall bar was characterized in a magnetic field at 1.5 K. Figure S1 represents the longitudinal resistivity ρ_{xx} of the device measured at three representative gate voltages: 0.3 V , 0.55 V and 0.69 V. The left column, Figure S1 (a), (c) and (e), illustrates ρ_{xx} in dark, while the right column, Figure S1 (b), (d) and (f), displays results after red-light illumination, applied until saturation of the transport parameters is achieved. All six plots in Figure S1, corresponding to different gate biases and illumination conditions, exhibit clean Shubnikov–de Haas oscillations. Fast Fourier transforms (FFTs) of the longitudinal resistances are analyzed to extract the electron density in the 2DEG. In all cases, only a single dominant peak is observed in the FFT spectrum, confirming the presence of a single 2DEG channel.

The transverse resistivity (or Hall resistivity) ρ_{xy} of the Hall bar measured in the dark across gate voltages ranging from 0.25 V to 0.69 V are illustrated in Figure S2. Clear quantization of the transverse resistivity along the increasing magnetic field is observed, proving that the wafer has a high sample quality.

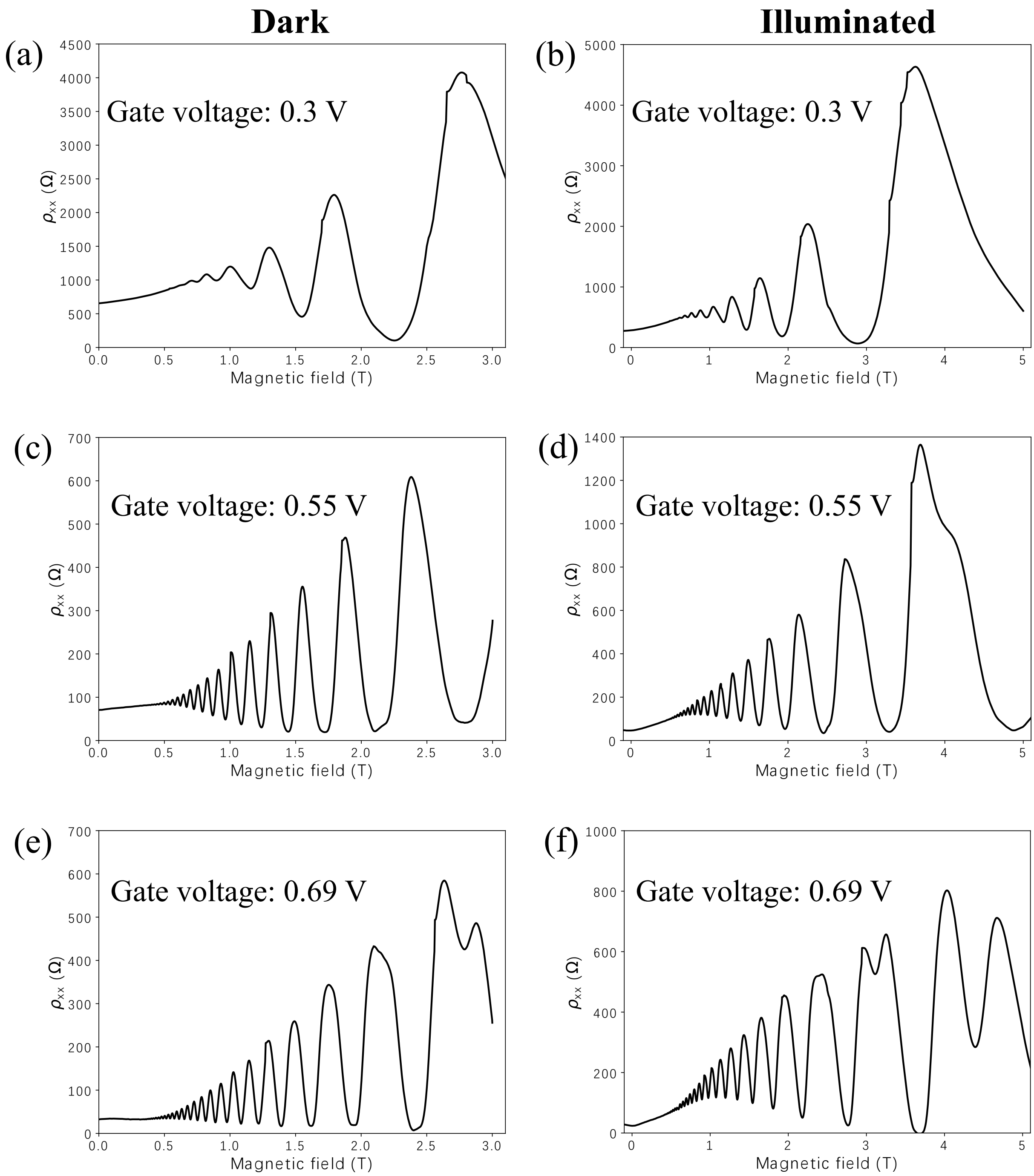

Figure S1. Longitudinal resistivity of the AlGaAs/GaAs wafer. Shubnikov–de Haas oscillations are observed for a set of gate voltage, 0.3 V, 0.55 V and 0.69 V, measured in dark (left column) and after red-light illumination (right column).

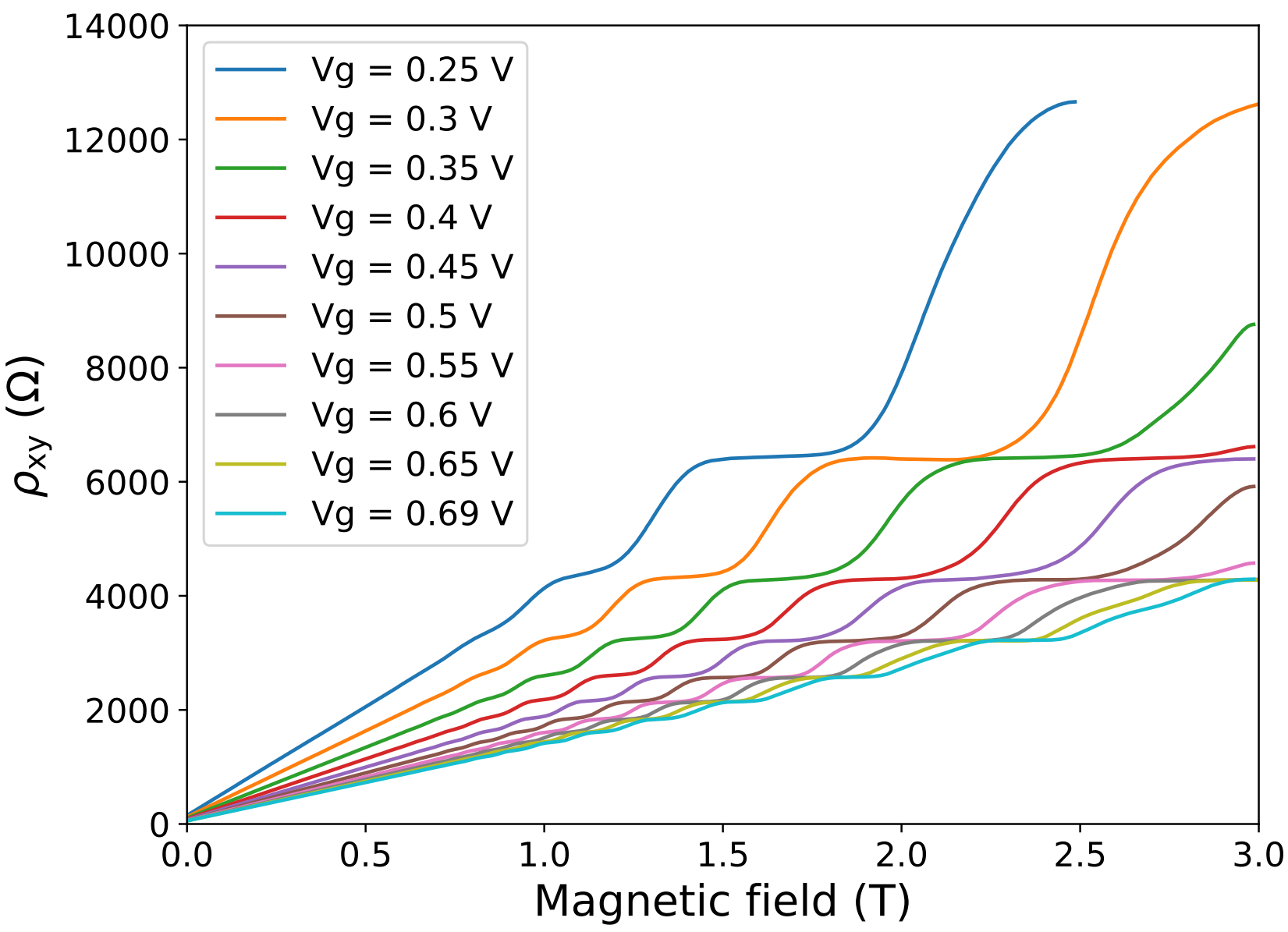

Figure S2. Transverse resistivity (or Hall resistivity) of the AlGaAs/GaAs wafer measured in the dark across gate voltages ranging from 0.25 V to 0.69 V.

The electron density within the 2DEG can be acquired from the Hall resistivity ρ_{xy} using Equation S1:

$$\rho_{xy} = \frac{B}{n_s e} = \frac{h}{\nu e^2}. \tag{S1}$$

Alternatively, it can be extracted from the FFT of Shubnikov–de Haas oscillations at low magnetic fields, as described by Equation S2:

$$\Delta\left(\frac{1}{B}\right) = \frac{1}{B_1} - \frac{1}{B_2} = \frac{e}{hn_s}. \tag{S2}$$

As illustrated in Figure S3 (a), the electron densities obtained by these two methods show good agreement. The single electron density values calculated from the FFT of the longitudinal resistivity ρ_{xx} indicates that only one 2DEG channel is present in the wafer.

According to the parallel-plate capacitor model, the 2DEG depth d can be estimated from the slope of the $n_s(U_G)$ data. The gate evaporated on top of the device and the underlying 2DEG channel can be treated as two metal 'plates'. The capacitance of the device is

$$C = \frac{\varepsilon_0 \varepsilon_r A}{d}, \tag{S3}$$

where d is the 2DEG depth, A is the area of the top gate, and ε_r is the relative dielectric constant of the material. With the capacitance formula

$$Q = en_s A = C(V_\mathrm{g} - V_\mathrm{th}), \tag{S4}$$

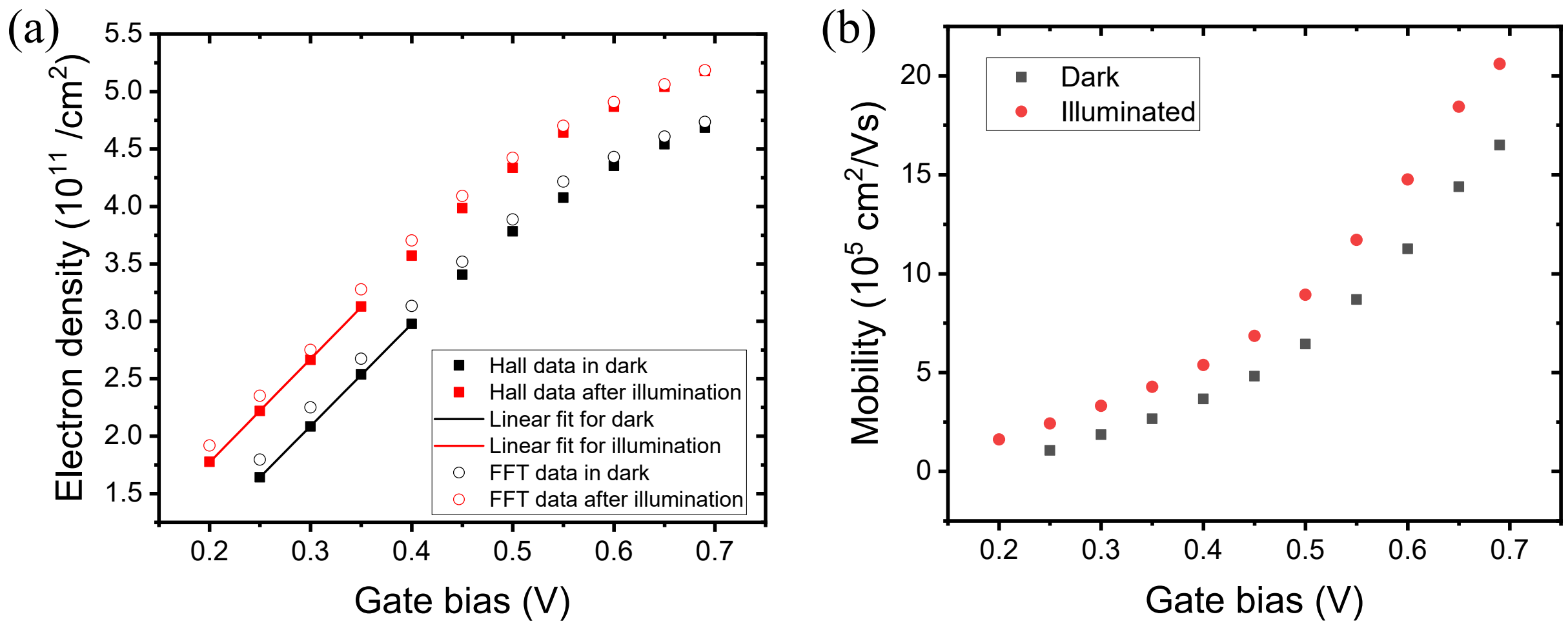

Figure S3. (a) Electron density and (b) mobility of the AlGaAs/GaAs wafer as a function of the gate voltage. The wafer is characterized both in the dark (black points) and after red-light illumination (red points). The electron density in (a) is calculated from both the Hall resistivity ρ_{xy} (marked by squares) and the FFT of Shubnikov–de Haas oscillations (marked by circles). The Hall data are fitted with linear functions to estimate the 2DEG depth using Equation S5. The mobility in (b) is derived from the Hall data using Equation S6.

the 2DEG depth d can be estimated as

$$d = \frac{\varepsilon_0 \varepsilon_r}{e} \cdot \frac{\Delta V_g}{\Delta n_s}. \tag{S5}$$

The $n_s(U_G)$ data from Hall resistivity are fitted with linear functions as illustrated in Figure S3 (a). This gives a 2DEG depth of $d = 77.4$ nm. The extracted value $d = 77.4$ nm is slightly larger than the geometrical layer thickness of 70 nm, which is expected considering that the wave function of the 2DEG in z-direction has a certain spatial extension.

The mobility of the 2DEG can be calculated using the equation

$$\mu_e = \frac{1}{n_s \, e \, \rho_{xx}(B=0)}. \tag{S6}$$

The extracted values of mobility in the dark and after red-illumination are shown in Figure S3 (b). The electron density $n_s(U_G)$ data from the Hall resistivity is utilized here for mobility estimation.

In summary, the magnetotransport results indicate that no obvious parallel conductance is present in the wafer, even under high gate bias and after red-light illumination. Only one 2DEG channel around 77 nm below the top surface is observed. The result is proved by the clean Shubnikov–de Haas oscillations with single dominant peaks in the FFT spectra.

2 Extended description of the experimental setup

An experimental setup is created to expose the PETS THz detector to THz radiation from the top, while illuminating it with an infrared LED from the backside, through the 500 µm substrate. The substrate-side illumination ensures homogeneous excitation of the 2D electron system, preventing a shadowing effect from the gates. As illustrated in Figure S4, the PETS THz detector is mounted on a copper plane, which is connected to the backside of a printed circuit board (PCB). The assembly is mounted onto a coldfinger. A 1-mm diameter hole throughout the copper support of the sample enables unobstructed access for illumination of the detector back side using an infrared LED with an IR centre-wavelength of $\lambda = 940\,\text{nm}$ ($E = 1.32\,\text{eV}$). THz radiation from the source in the setup, a quantum cascade laser (QCL)[S2] with a wavelength of $\lambda = 159.38\ \mu\text{m}$ (1.881 THz emission frequency), is delivered to the front of the detector.

Figure S5 shows the sample holder used in this work. The PETS detector in the centre is mounted on top of a 1-mm hole.

An OSRAM IR OSLON® Black Series LED[S3] with a centre wavelength of $\lambda = 940\,\text{nm}$ ($E = 1.32\,\text{eV}$) was employed to illuminate the PETS THz detector from its backside. As illustrated in Figure S6 (a), the infrared LED was glued into the cap of the detector cryostat using an Electrolube Thermal Bonding System (TBS)[S4] to ensure adhesion and high thermal conductivity. Electrical bias was supplied to the LED via two holes drilled into the cryostat cap. Figure S6 (b), captured with an infrared viewer, shows the IR LED in operation.

The Light-current-voltage (LIV) curves of the LED were characterized (Figure S7) to deliver a controlled, quantifiable illumination dose during the experiment. The distance between the power meter and the LED was set equal to that between the sample and the LED in the experimental setup, to accurately determine the optical power incident on the PETS THz detector.

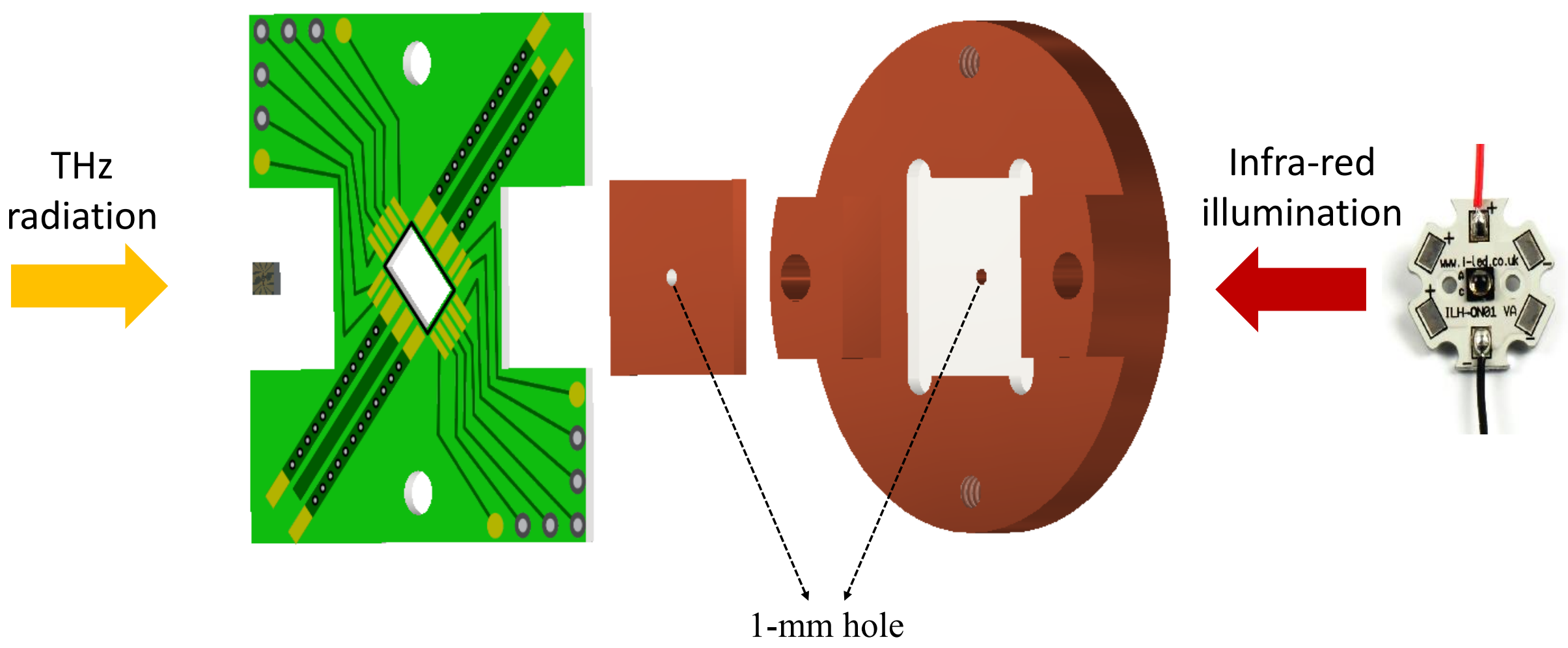

Figure S4. Setup for back-side infrared illumination. Components included in the setup, from left to right, are a PETS THz detector, an RF PCB, the insert of the copper holder with a 1-mm hole at the centre, the nest of the copper holder with a 1-mm hole at the centre, and an infrared LED with an IR centre-wavelength of $\lambda = 940\,\text{nm}$. THz radiation is delivered to the front side of the device, and the infrared LED illuminates the back-side of the detector.

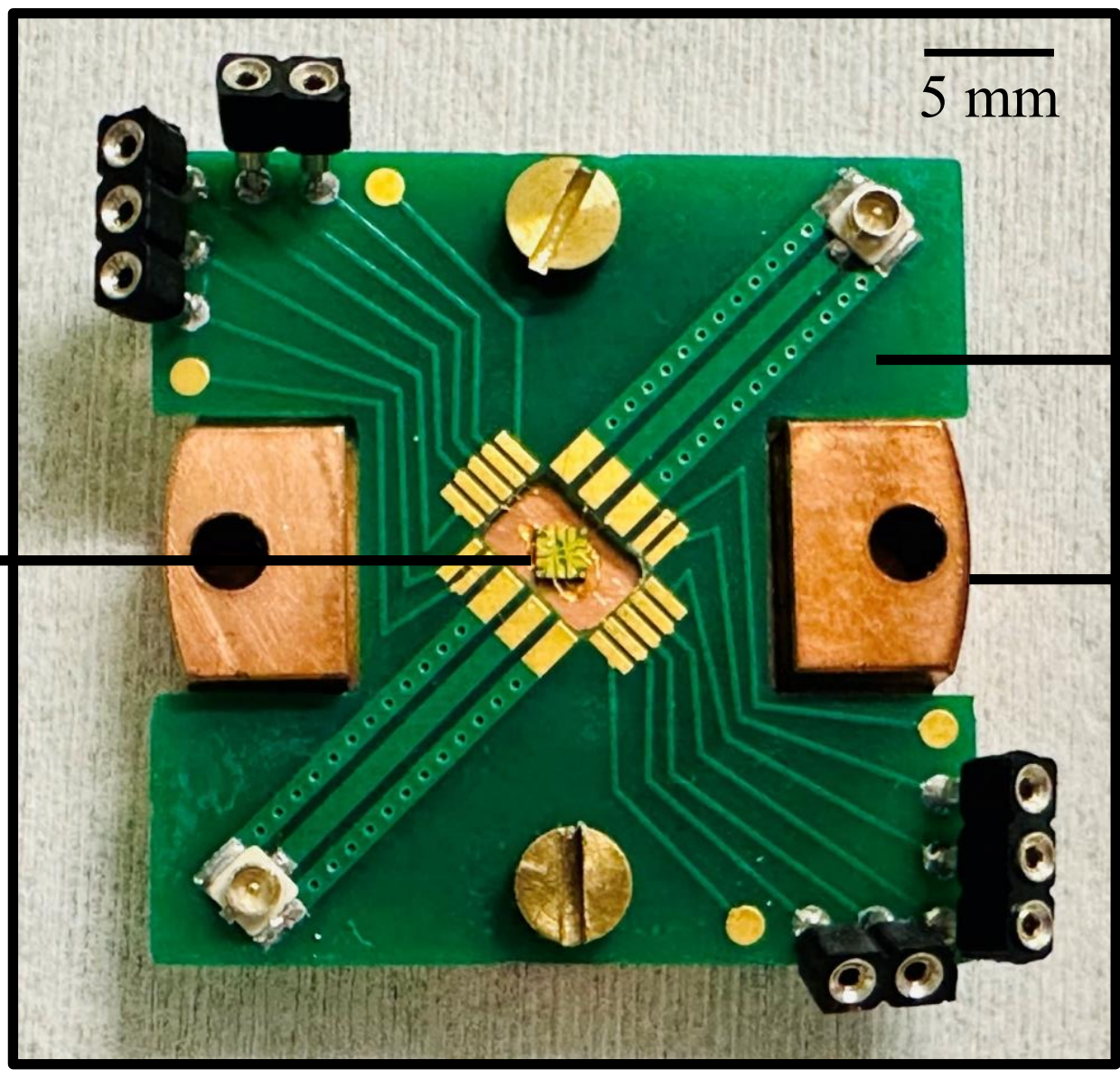

Figure S5. Photo of the sample holder. A PETS THz detector is mounted on a copper holder and bonded onto the PCB.

A 1-mm iris was placed in front of the optical power meter to replicate the radiation received by the detector via a 1-mm hole in the copper holder.

As illustrated in Figure S7 (a), with the 1-mm iris, the radiation intensity from the LED increases linearly with an increasing current until $\sim 930\,\mathrm{mA}$; after that it starts to exceed the limit of the power meter. By contrast, the L-V curve is non-linear, as shown in Figure S7 (b). The LED does not emit detectable radiation until the forward voltage reaches $\sim 2\,\mathrm{V}$. To reach larger illumination doses shown in the figures in the main manuscripts, we have used forward currents larger than 1-mA, but the resulting illumination dose has been normalised to the incident power at 1-mA forward current.

3 Reproducibility of the infrared illumination-induced response

The infrared illumination-induced behaviors demonstrated in the main manuscript were reproduced over multiple independent cool-down and measurement cycles. Results from an additional independent cool-down are presented in Figures S8 and S9 as a representative example, further confirming the reproducibility of the observed behaviors.

Under the same experimental conditions as those described in the main text, the device was measured at $\sim$ 6 K with the wide gate biased at 0.75 V. 940-nm infrared radiation from a LED illuminated the PETS THz detector from the backside through a 1-mm diameter hole. The "illumination time" refers to the cumulative IR illumination dose, normalized to a unit energy dose of 20.24 μJ.

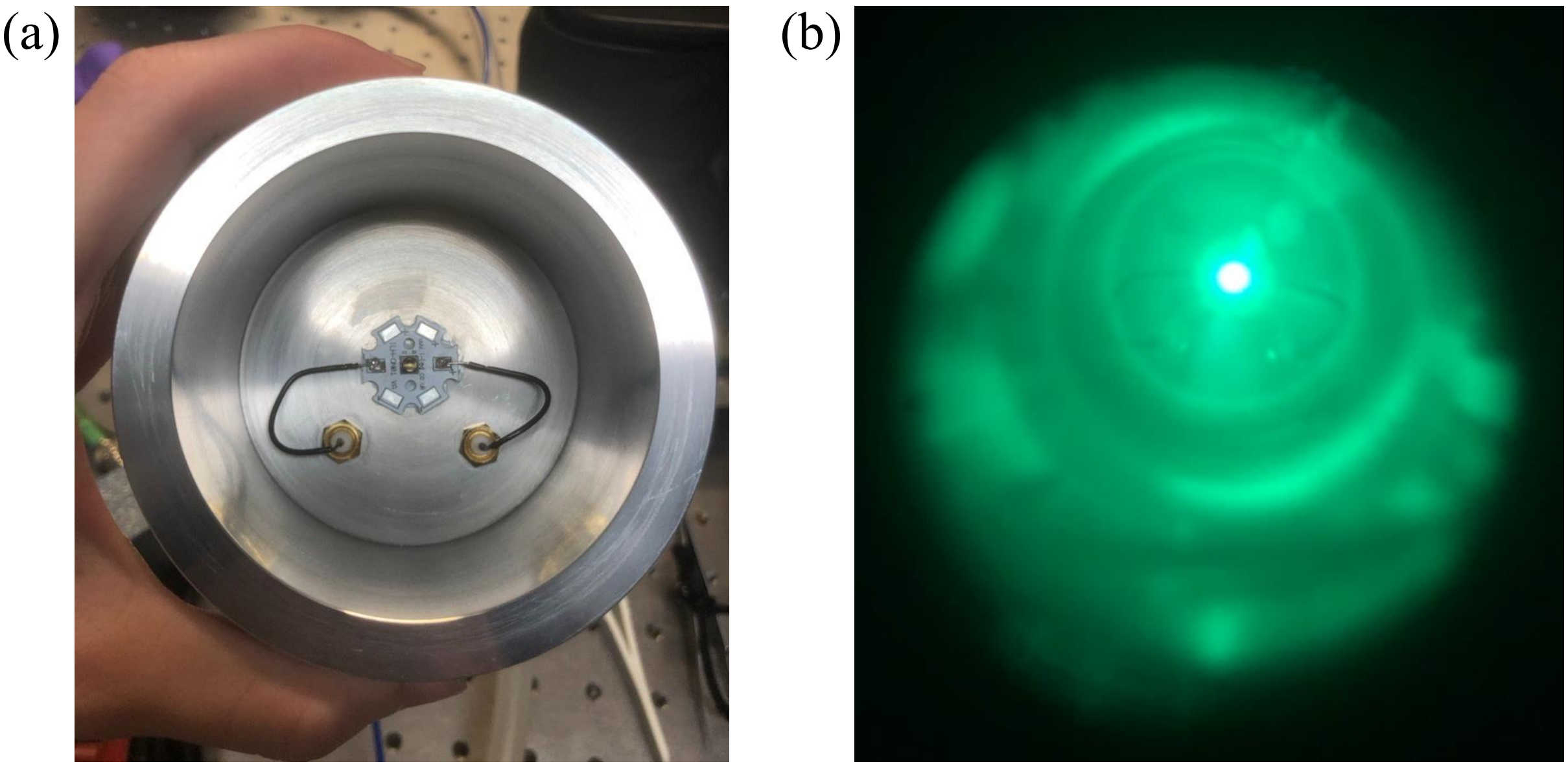

Figure S6. Infrared LED stuck into the cap of the detector cryostat. (a) A photo of the interior of the cap with a LED glued inside. The LED is off in this image. (b) A photo taken through an infrared viewer with the LED on.

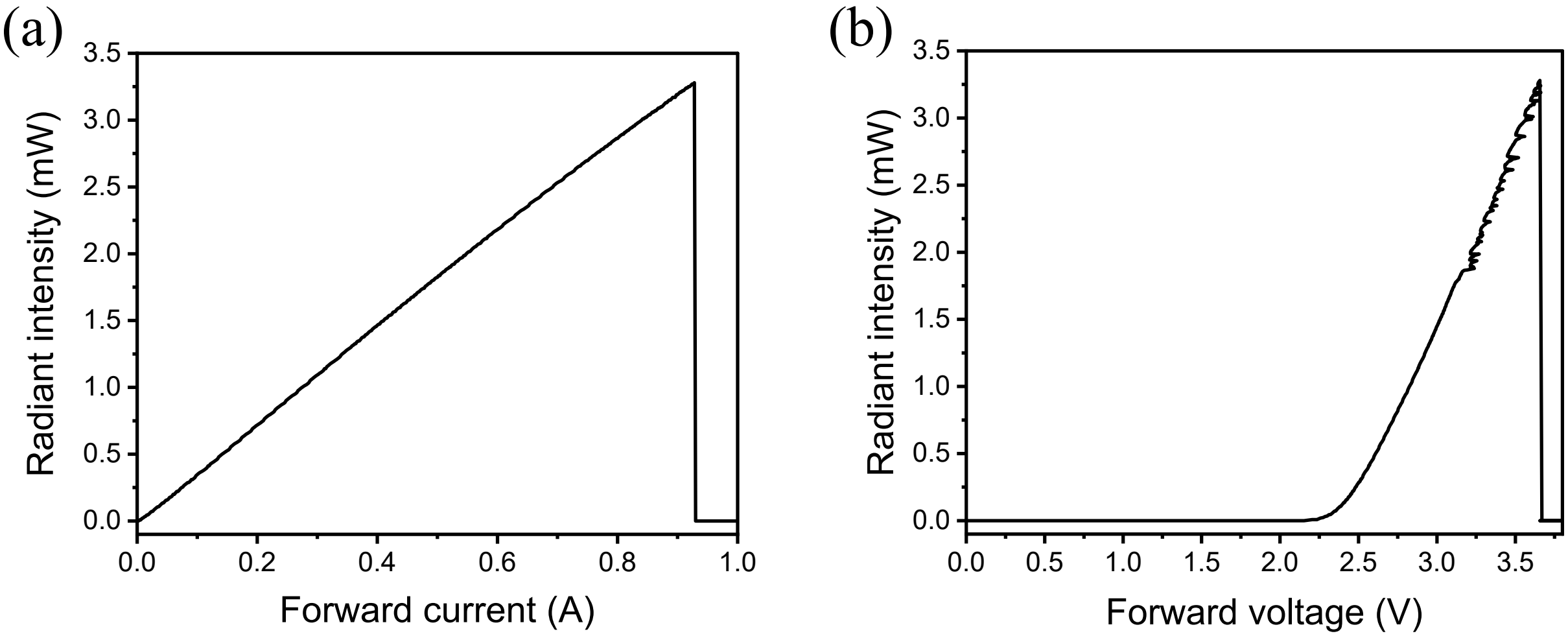

Figure S7. LIV curves of the infrared LED. (a) L-I curves and (b) L-V curves of the IR LED with a 1-mm iris at the front.

A similar characteristic dependence on infrared illumination is observed in the independent measurement. The photocurrent response (Figure S8(a)) increases with continued illumination until saturation, with a sudden shift of the narrow gate voltage for $\max(I_{ph})$ in the hundreds-of-seconds regime. Under continued illumination, the photovoltage response (Figure S8(b)) rises up slightly at the beginning, followed by a small decrease after saturation, accompanied by a sudden shift in the narrow gate voltage for $\max(V_{ph})$ on the timescale of hundreds of seconds. In good agreement with the change of photocurrent and photovoltage responses, the conductance of the 2DEG channel (Figure S8(c)) increases significantly with infrared illumination. The wide gate leakage at 0.75 V (Figure S8(d)) is also consistent with Figure 5(b) in the main manuscript:

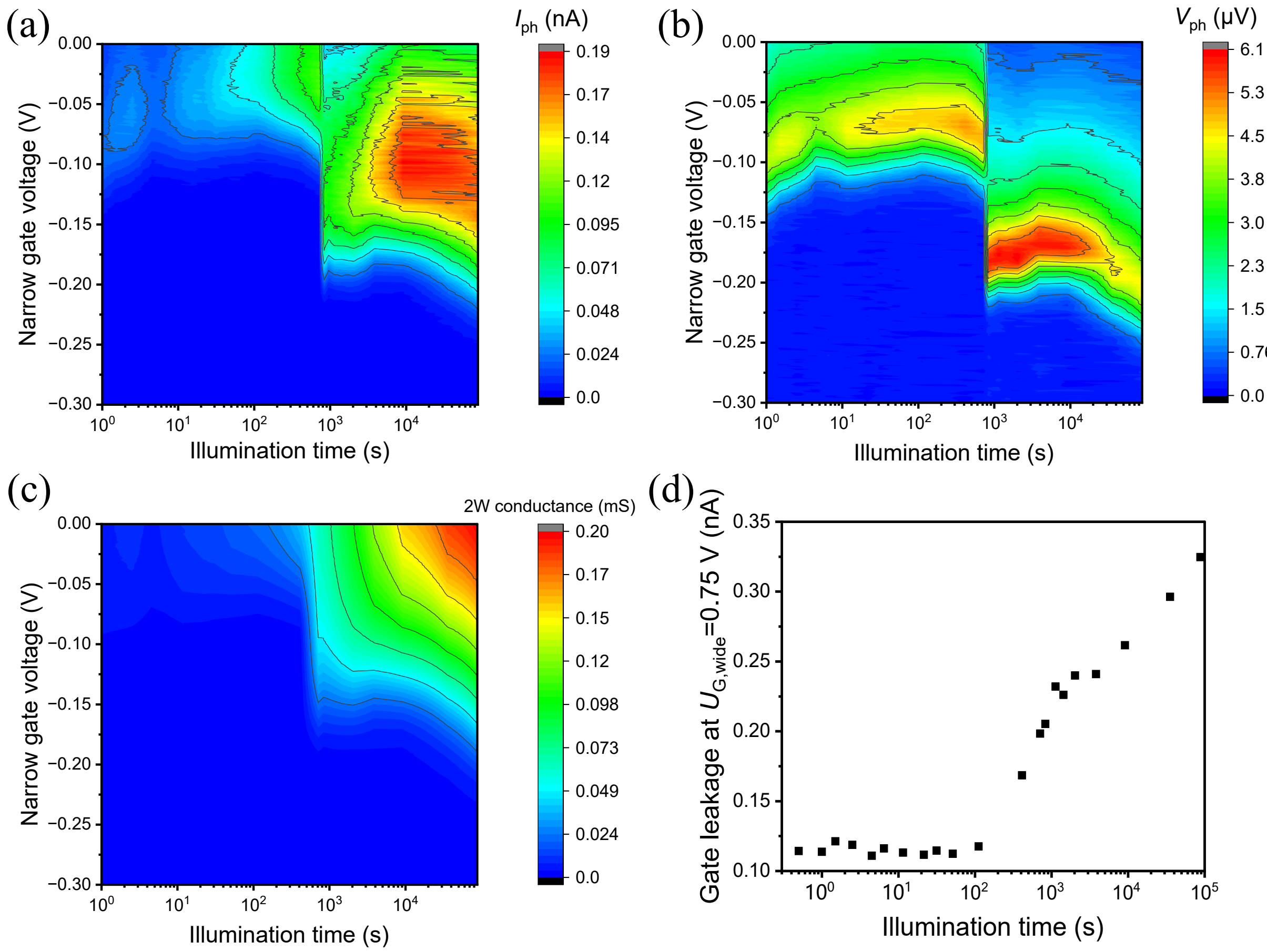

Figure S8. Reproducibility measurements from an independent cool-down of the AlGaAs/GaAs-based PETS THz detector under IR illumination. (a) Photocurrent response, (b) photovoltage response, and (c) two-terminal conductance as functions of the narrow gate voltage and the accumulated illumination time. The measurements were performed at approximately 6 K with the wide gate biased at 0.75 V. (d) Leakage current of the wide gate biased at 0.75 V, in dependence on IR illumination time.

it remains constant initially and then gradually increases after $\sim$ 200 s. Despite this change, the gate leakage is negligible throughout the entire illumination period due to its small magnitude.

The threshold voltage (U_{th}) and the corresponding equilibrium electron density (n_0) are also consistent with the results presented in Figure 5(a) of the main manuscript. U_{th} obtained by fitting the conductance curves using Equation 2 in the main manuscript, shifts from $\sim$ -0.1 V to $\sim$ -0.2 V in the hundreds-of-seconds regime as shown in Figure S9 (a). n_0 is estimated using the parallel-plate capacitor model with Equation 1 in the main manuscript. As shown in Figure S9(b), with accumulated IR illumination, n_0 initially decreases slightly, then rises up steeply to a plateau, followed by a gradual further increase.

Compared with the results presented in the main manuscript, the maximum IR illumination dose applied in this measurement ($< 10^5$ s) lies below the dose resulting in a saturation of the device. Therefore, the saturation behavior of n_0 and U_{th} observed in Figure 5 of the main manuscript, where the device was illuminated for 10^6 s, is not seen in this measurement.

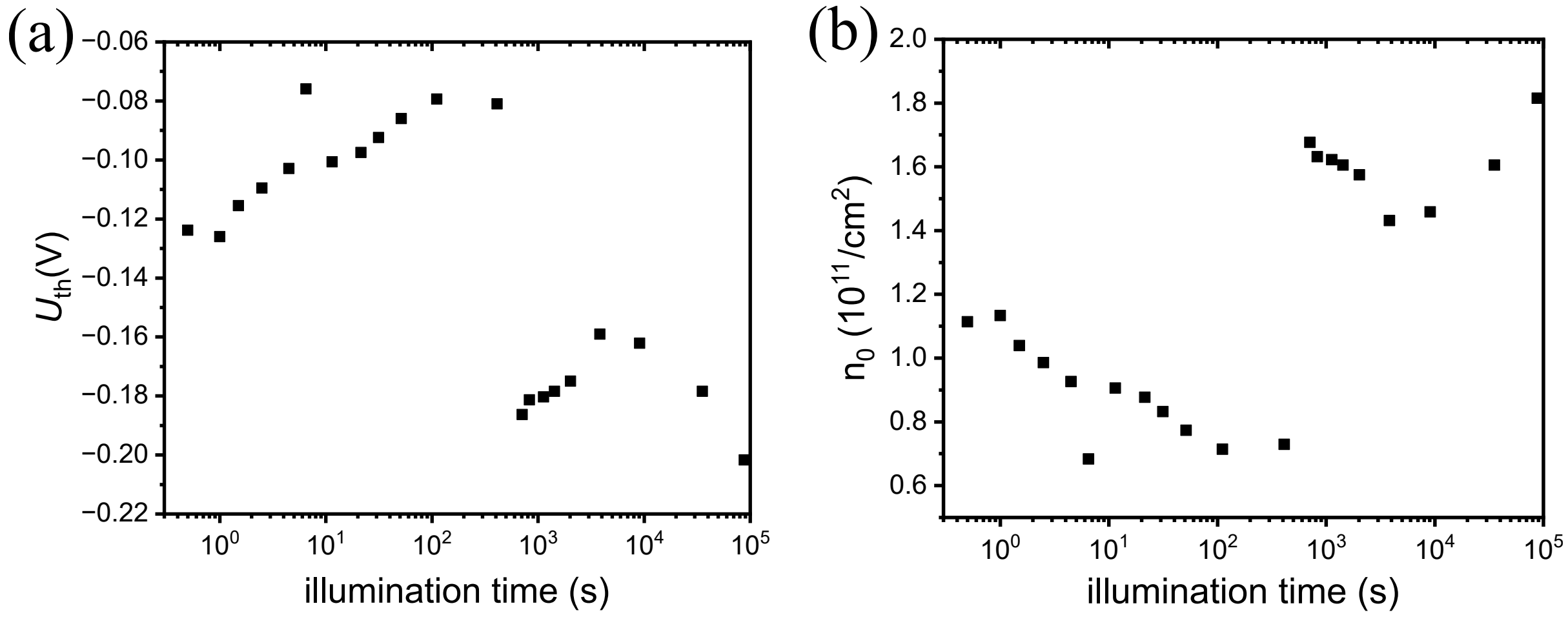

Figure S9. Threshold voltage and equilibrium charge carrier density extracted from the independent cool-down measurements shown in Figure S8. (a) Threshold voltage U_{th} as a function of the illumination time. U_{th} are obtained by fitting the two-terminal conductance curves (Figure S8(c)) using Eq. (2) in the main manuscript. (b) The corresponding equilibrium charge carrier density n_0 calculated from U_{th} using Eq. (1) in the main text.

References

(S1) Waldrop, J. Schottky-barrier height of ideal metal contacts to GaAs. *Applied physics letters* **1984**, *44*, 1002–1004.

(S2) Worrall, C.; Alton, J.; Houghton, M.; Barbieri, S.; Beere, H. E.; Ritchie, D.; Sirtori, C. Continuous wave operation of a superlattice quantum cascade laser emitting at 2 THz. *Optics Express* **2006**, *14*, 171–181.

(S3) Intelligent LED Solutions. OSLON® Black 1 PowerStar IR ILH-I#01-####-SC2#1-WIR200. Series. https://docs.rs-online.com/1ae5/0900766b81411828.pdf (accessed 08/20/2026).

(S4) Electrolube. TBS Thermal Bonding System. https://electrolube.com/app/uploads/2019/09/TBS.pdf (accessed 08/20/2026).
